# Synthesis and photochemical response of Ru(ii)-coordinated double-decker silsesquioxane†

**DOI:** 10.1039/c7ra12290j

**Published:** 2018-01-09

**Authors:** Asuman Celik Kucuk, Jun Matsui, Tokuji Miyashita

**Affiliations:** Metallurgical and Materials Engineering, Marmara University Gostepe Campus 34722 Istanbul Turkey asuman.celik@marmara.edu.tr; Department of Material and Biological Chemistry, Yamagata University 1-4-12, Kojirakawamachi Yamagata 990-8560 Japan jun_m@sci.kj.yamagata-u.ac.jp; Institute for Multidisciplinary Research for Advanced Materials, Tohoku University 2-1-1, Katahira, Aoba-ku Sendai 980-8577 Japan

## Abstract

A double-decker silsesquioxane based bis(terpyridine) ruthenium(ii) complex (2Tpy/Ru-DDSQ), a member of the polyhedral oligomeric silsesquioxane (POSS) class, has been synthesized. Its structure has been characterized using comprehensive techniques such as nuclear magnetic resonance (^1^H NMR) spectroscopy, X-ray photoelectron spectroscopy (XPS), and UV-visible spectroscopy. This work not only deals with the synthesis of 2Tpy/Ru-DDSQ but also provides the first comprehensive investigation based on the photoluminescence and electrochemical features of a POSS member. With this structure, an efficient anodic photocurrent response has been obtained. This result has been attributed to the perfect arrangement of ruthenium(ii)-bis(terpyridine) (tpy) ([Ru(Tpy)_2_]^2+^) moieties in the DDSQ nano building blocks. Therefore, 2Tpy/Ru-DDSQ can be considered as a promising candidate for the development of new generation photosensitizers.

## Abstract 2

A double-decker-silsesquioxane (DDSQ) based transition-metal-complex showed efficient energy-conversion owing to the assembling ability of the complexes in the DDSQ nano-building blocks.

## Graphical abstract

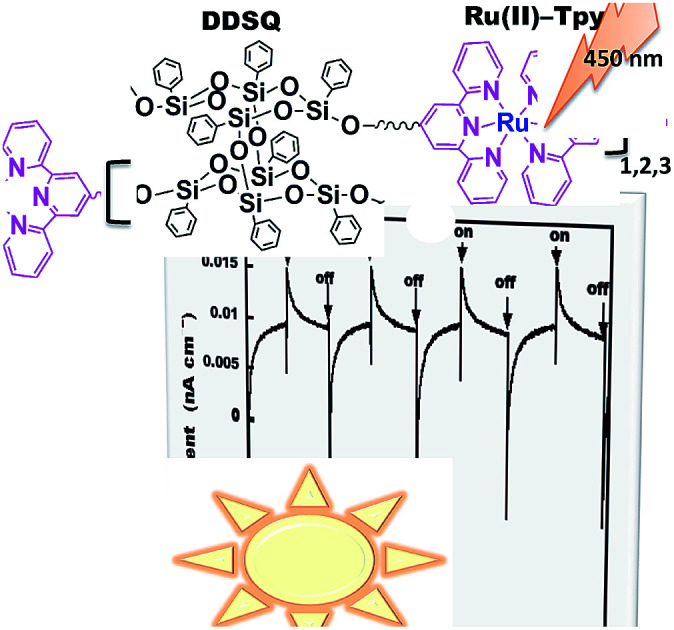

## 1. Introduction

The arrangement and stability of transition-metal complexes play key roles in achieving high-efficiency in light-to-electricity conversion systems.^1–3^ Since coordinate bonds provide extended networks with well-defined geometries, developing transition metal based supramolecular coordination complexes with high thermal, chemical and structural stability as well as structural regularity and controllability^4,5^ is a promising approach. This approach is very helpful especially in the study of photoinduced electron- and energy transfer processes.^6^ However, supramolecular materials are known for their limited solubilities.^7^ Since telechelic monomers are capable of self-condensation with transition metal ions, the solubility problems of the supramolecular materials could be overcome by using appropriate telechelic monomers.^8^

Cubic polyhedral oligomeric silsesquioxanes (POSSs) have attracted much attention to be used as supramolecular nano building blocks.^9,10^ A POSS molecule consists of a rigid inorganic SiO_1.5_ core surrounded by multiple tunable organic coronae (R_2_SiO). Therefore, it can be referred as hybrid material.^11^ Core with a nanometer scale provides higher homogeneity, reproducibility and predictability. Additionally, coronae give opportunities to tailor some properties of the molecules such as reactivity and solubility.^12^ Owing to the extraordinary properties of POSS, the attempts on using POSS derivatives as a part of transition metal based supramolecular coordination complex have been gradually increasing. In a previous study, POSS derivatives used as central cores of dendrimer to form highly functionalized molecular systems. Obtained metallo-dendrimer with Ru(ii)-based chromophore exhibits summation effects in the electronic absorption measurements as well as an improvement in the quantum yield.^13^ Another study by Yam V. W. *et al.* has reported platinum(ii)-terpyridine (Tpy) complex incorporated into POSS moieties. This study demonstrated that self-association process was governed by hydrophobic interactions owing to the POSS moieties. Morphological transformation of POSS moieties depending on the solvent media resulted in some interesting spectroscopic changes.^14^ Another study by Koytepe S. *et al.* reported POSS possessing terminal terpyridine (Tpy) ligands and they developed stable POSS coordination complexes with Cu or Co.^15^ On the other hand, Akira Endo *et al.* managed the synthesizing a long-range ordered coordination polymer using POSS with eight terminal groups as a ligand through the controlling of reaction temperature.^16^ Lastly, Esther Carbonell *et al.* managed to synthesis three-dimentional coordination polymer. They used POSS decorated with eight terpyridine moieties and investigated its complexes with two different metal (Zn^2+^ and Fe^2+^) by absorption and emission spectroscopy.^17^ Although substantial amount of researches on transition metal complexes of POSS are available in the literatures, none of them is about their affects on photoelectrochemical features.

It is well known that conventional POSS has a rigid structure and at least eight reactive sites. Thus, it is difficult to functionalize and to purify it. Moreover, rigid structure causes aggregation. Double-decker shaped silsesquioxane (DDSQ), one of the members of POSS family, has some structural differences that bring additional advantages such as high yield, easy purification, and relatively less rigidness.^18^ On the other hand, it has been found that ability to form “core-coronae” type amphiphiles reduces aggregation tendencies of DDSQ molecules^18^ and increases the solubility.^19–21^ Thus, using DDSQ derivatives as telechelic monomers may increase the solubility of supramolecular coordination complex and overcome the difficulties on conjugating of POSS with the metal centres.

In order to investigate the affects of a POSS member on the photochemical properties, DDSQ based Ru(ii)-bis(terpyridine) complex (2Tpy/Ru-DDSQ) has been synthesized. Characterization of 2Tpy/Ru-DDSQ has been carried out by using Fourier transform IR (FT-IR), nuclear magnetic resonance (^1^H NMR) spectroscopy, X-ray photoelectron spectrometer (XPS), UV-visible spectroscopy and cyclic voltammogram (CV). 2Tpy/Ru-DDSQ is found to be electrochemically stable and generates efficient photocurrent due to the good assembling ability of the [Ru(Tpy)_2_]^2+^ moieties in the DDSQ nano building blocks.

## 2. Experimental

### 2.1 Materials

DDSQ containing four epoxy groups (4EPX-DDSQ) were kindly donated by JNC Corp. Toluene, dimethyl sulfoxide (DMSO) and acetonitrile were purchased respectively from Nacalai Tesque Inc. They were used without further purification. Platinum divinyl-tetramethyldisiloxane (Pt(dvs), 3 wt% in xylene solution) was obtained from Umicore. Dichloromethane, chloroform, hexane, acetone, methanol (MeOH), and ethyl ether were purchased from Kanto Chemical Co. Inc. and distilled before use. Spectroscopic grade chloroform (Dojindo Laboratories) was used as a casting solvent. Anhydrous tetrahydrofuran (THF), RuCl_3_·3H_2_O, 4′-chloro-2,2′:6′,2′′-terpyridine and 2,2′:6′,2′′-terpyridine compounds were purchased from Aldrich Chemical Co. Inc. and used as received.

### 2.2 General methods

FT-IR spectra were obtained using an FT-IR spectrometer (FTIR4200; Jasco Inc.). IR spectra of DDSQ based materials were recorded between 4000 and 750 cm^−1^ with 4 cm^−1^ resolution, under a continuous nitrogen purge. ^1^H and ^13^C NMR measurements were carried out with a JEOL JNM-AL 400 spectrometer, in CDCl_3_ or DMSO-*d*_6_ without tetramethylsilane. For MALDI-TOF MS (Bruker Daltonics) analysis, the matrix 1,8,9-anthracenetriol (dithranol) (10 mg) and the salt silver trifluoroacetate (AgTFA) (2 mg) was separately dissolved in THF (400 μL). 2 mg of sample (100 μmol in THF) was dissolved in 100 μL THF. The MALDI analysis sample was prepared as following three-step droplet method. The resultant solutions with the volume ratio of 1 : 1 : 1, v/v/v were separately deposited on a stainless steel sample plate and dried. The measurement was done in linear mode with a N_2_ laser (337 nm), and positive mode with an accelerating voltage 20 kV. A X-ray photoelectron spectrometer (XPS, PHI 5600; PerkinElmer Inc.) was used to control elemental composition and oxidation state of elements at the surface. All binding energies in XPS measurements were referenced to the C 1s peak for neutral carbon, which was assigned as a value of 285.0 eV. The take off angle was fixed at 45°.

### 2.3 Spectroscopy and photocurrent measurements

UV-vis absorption spectra were measured using a Hitachi U-3000 UV-vis absorption spectrometer. Emission spectra were measured using a Hitachi F-4500 spectrofluorophotometer. The analyses were carried out using the home-made cell (ESI, Fig. S11†). An ITO electrode was cleaned respectively by sonication in isopropyl alcohol, chemical detergent solution, and distilled water. Then ITO was modified with 2Tpy/Ru-DDSQ and used as a working electrode. Silicone rubber (width × length × height = 20 mm × 20 mm × 6 mm) was placed onto working electrode to create a rectangular well and 0.1 M KClO_4_ aqueous electrolyte that was bubbled with nitrogen gas for 30 min prior to the photocurrent measurement was poured into the space. Then, the well was covered by glass substrate. A platinum wire and Ag/AgCl were used as counter and a reference electrode, respectively. The wires were put in the cell through the silicone rubber. The photocurrent was measured using a potentiostat (ALS 611B; BAS, Inc.). Two xenon lamps (500 W) equipped with UV cutoff filters (VY43, Toshiba, black 50), IR cutoff filters (IRA-2S; Toshiba Corp.), and colour filters (at 450 nm, Y44, Yellow 440) were used to obtain the monochromic light. In these conditions, the radiant power landing on the surface of the cell window is 2 mW cm^−2^. During this experiment irradiation was alternatively switched on and off using a shutter. All measurements were carried out at room temperature. Data were smoothed with a locally weighted least squares method in Kaleidagraph (v. 4.2) software (SYNERGY SOFTWARE).

### 2.4 Synthesis of amine functional terpyridine, Tpy-NH_2_

6-Aminohexyl 4′-(2,2′:6′,2′′-terptridinyl) ether (Tpy-NH_2_) was prepared according to literature procedures.^22^ Briefly, to a stirred suspension of powdered KOH in dry DMSO at 30 °C, 6-amino-hexan-1-ol was added. After 30 min, 4′-chloro-2,2′:6′,2′′-terpyridine (Tpy-Cl) was added, and then the mixture was stirred for 4 h at 60 °C and then precipitated dropwise into the 0.5 L of ice-cooled, distilled water. After 2 h stirring, the precipitate was collected and washed with distilled water. The pure product was obtained in a 95% yield (ESI Scheme S1a†).

#### 2.4.1 Tpy-NH_2_

^1^H NMR (ppm) (CDCl_3_, 400 MHz): *δ*_H_ 8.69 (2H, d, Tpy6-6′′), 8.60 (2H, d, Tpy3,3′′), 8.00 (2H, s, Tpy3′,5′), 7.84 (2H, t, Tpy4,4′′), 7.36 (2H, t, Tpy5,5′′), 4.20 (2H, –CH_2_O–), 2.60 (2H, NH_2_CH_2_–), 1.85 (2H, –CH_2_CH_2_O–), and 1.50 (6H, –CH_2_CH_2_CH_2_–).

### 2.5 Preparation of amine functional bis(terpyridine) Ru(ii) complex; Tpy/Ru

Complexation reaction of Tpy-NH_2_ with RuCl_3_·3H_2_O was preceded and 6-aminohexyl 4′-(2,2′:6′,2′′-terpyridinyl) ether Ru(ii) complex (Tpy/Ru) was obtained as seen in the ESI Scheme S1b.† The reaction procedure is as following. A solution of Tpy-NH_2_ in methanol was stirred at 110 °C. Then equimolar amount of RuCl_3_·3H_2_O was added. Stirring was continued overnight. At the same temperature, equimolar amount of 2,2′:6′,2′′-terpyridine compound was added to the solution mixture. The resulting dark orange precipitate was collected by filtration and washed twice with ice-cold water, followed by the ethyl ether.

#### 2.5.1 Tpy/Ru

UV-vis absorption spectrometer (Hitachi U-3000): 280–300 nm; [π → π*] transition of alkynyl (phenyl groups attached to the DDSQ core) and Tpy ligand. 400–600 nm; the metal-to-ligand charge-transfer (MLCT) for [Ru(Tpy)_2_]^2+^.

### 2.6 Synthesis of telechelic DDSQ-Tpy monomer, 2Tpy-DDSQ

**Scheme 1:**
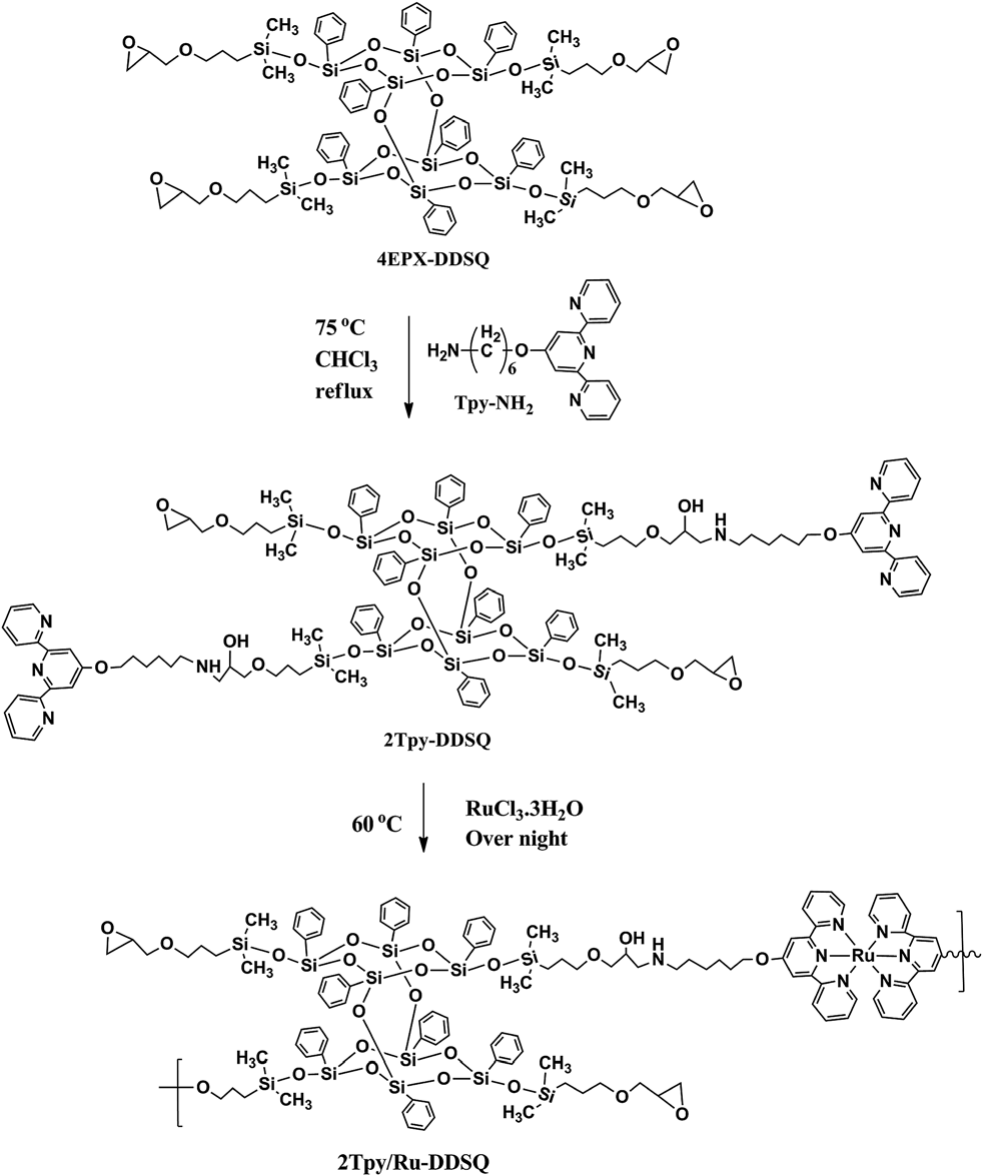
Synthesis of double-decker silsesquioxane based bis(terpyridine) Ru(ii) complex (2Tpy/Ru-DDSQ).

Epoxy-amine curing reaction was proceeded according to the literature procedures.^23,24^ Both reactants 4EPX-DDSQ^25^ (1.15 g, 0.65 mmol) and 2,2′:6′,2′′-terpyridine (Tpy-NH_2_; 1 g, 2.9 mmol) were dissolved in THF and then reaction mixture heated till 75 °C step by step. The solvent was evaporated. The pure product was obtained in a 20% yield after column chromatography (with THF/hexane eluent system). The possible products obtained from the reaction of 4EPX-DDSQ with Tpy-NH_2_ have been displayed with their exact molecular weight in ESI Chart S1.† Estimated molecular weights of possible products after a single reaction of the primer amine with an epoxy group are as in the following; 2104 g mol^−1^ for 1Tpy-DDSQ, 2452 g mol^−1^ for 2Tpy-DDSQ, 2801 g mol^−1^ for 3Tpy-DDSQ and 3149 g mol^−1^ for 4Tpy-DDSQ (ESI Chart S1†). Theoretical molecular weight of 2Tpy-DDSQ in which two Tpy ligands connect to one component of DDSQ core, is 2452. Therefore, the base peak at *m*/*z* = 2450.59 [M − 2] can be associated with the molecular ion peak of 2Tpy-DDSQ. The results from MALDI-TOF/MS and ^1^H NMR have shown that reaction has been processed through two arms of DDSQ (Scheme 1). Therefore 2Tpy-DDSQ in which two Tpy ligands connected to one DDSQ core has been considered as the yield of reaction (Scheme 1). It is believed that Tpy-NH_2_ molecules have been attached to ethylene oxide rings of the DDSQ molecule that are in the diagonal position due to the steric hindrance effect of Tpy ligands.

The difference between theoretical molecular weight and base peak is probably attributed to loss of two hydrogen atoms shared by two nitrogen atoms maybe after ionisation process of MALDI-TOF/MS. It is well known that in the positive ion mode, compound generally has been positively charged. However in some condition, for example in the basic solution, compound can be negatively charged even in the positive mode.^26^ Although MALDI-TOF/MS of 2Tpy-DDSQ has been done in positive mode, 2Tpy-DDSQ has been negatively charged. Analysing molecular weight of analyte has been carried out under the presence of 1,8,9-anthracenetriol (dithranol) as a matrix and AgTFA as a salt. The negatively charged manner in the positive mode may be attributed to the some interaction of OH or NH groups at the chains' of DDSQ with environment.

#### 2.6.1 2Tpy-DDSQ

FT-IR (cm^−1^), *ν* = 3373 (broad, O–H), *ν* = 3297 (N–H), *ν* = 3100–2870 (aliphatic and aromatic C–H), *ν* = 1606, 1567 (Tpy aromatic H), *ν* = 1050 (Si–O), *ν* = 1150 (Si–C). ^1^H NMR (ppm) (CDCl_3_, 400 MHz): *δ* (ppm) 8.69 (2H, d, Tpy6-6′′), 8.60 (2H, d, Tpy3,3′′), 8.00 (2H, s, Tpy3′,5′), 7.84 (2H, t, Tpy4,4′′), 7.36 (2H, t, Tpy5,5′′), 4.20 (2H(f), m), 3.25–2.92 (5H(i, j, h), m), 2.51 (2H(g), m), 1.81 (2H(a), m), 1.81–1.21 (10H(b, c, d, e, k), m), and 0.37 (2H(l), m). MALDI-TOF MS = 2450 (M − 2, calculated value 2452).

### 2.7 Synthesis of DDSQ based bis(terpyridine) Ru(ii) complex, 2Tpy/Ru-DDSQ

A solution of 2Tpy-DDSQ in methanol (MeOH, 5 ml) and two fold excess amount of RuCl_3_·3H_2_O in MeOH (5 ml) was stirred for 30 min, heating under reflux. A few drops of *N*-ethylmorpholine were added and the solution turned from orange to reddish. Heating under reflux was continued overnight, after which an excess of NH_4_PF_6_ (45 mg, 0.27 mmol) was added. Subsequently, the reaction mixture was a cooled to 0 °C. The resulting dark orange precipitates were collected by filtration and washed twice with ice-cold water, followed by the ethyl ether.

The precipitates cannot be dissolved in THF, CHCl_3_, MeOH, hexane, CH_3_CN (Scheme 1). On the other hand, the precipitates can be dissolved in DMSO and acetone. The obtained structure was characterized by FT-IR, ^1^H NMR, XPS and UV visible spectroscopy. The mole fraction of Ru(tpy)_2_^2+^ was determined to be 0.22 from the UV-vis absorption spectrum (see ESI Fig. S10, Appendix part†).

#### 2.7.1 2Tpy/Ru-DDSQ

^1^H NMR (400 MHz, DMSO): *δ* (ppm): 8.85 (2H, t, Tpy3′,5′), 8.59 (2H, m, Tpy3-3′′), 7.79 (2H, s, Tpy4,4′′), 7.30 (2H, m, Tpy6,6′′), 7.28 (2H, t, Tpy5,5′′), 4.20 (2H(f), m), 3.25–2.92 (5H(i, j, h), m), 2.51 (2H(g), m), 1.81 (2H(a), m), 1.81–1.21 (10H(b, c, d, e, k), m), and 0.37 (2H(l), m). ^13^C NMR (100 MHz, DMSO): *δ* (ppm) 166.40, 165.42, 158, 77, 158.72, 158, 29, 155.92, 153.10, 152.53, 152.35, 138.45, 128.53, 125.14, 124.95, 111.89, 86, 07, 70.46, 42, 78, 42.53, 41.00, 35.13, 34.64.

## 3. Results and discussion

Synthesis of DDSQ based bis(terpyridine) Ru(ii) complex has two stages: first stage is the synthesis of the telechelic DDSQ-Tpy monomer whereas the second stage is the synthesis of DDSQ based Ru(ii)-Tpy coordination complex. Each stage has been explained in detail. Then, photocurrent properties of DDSQ based bis(terpyridine) Ru(ii) complex have been investigated.

### 3.1 Synthesis and characterization of the telechelic DDSQ-Tpy monomer, 2Tpy-DDSQ

6-Aminohexyl 4′-(2,2′:6′,2′′-terpyridinyl) ether compound (Tpy-NH_2_) has been synthesized as in the literature and utilized as a curing agent for modification of DDSQ. ^1^H NMR has been used for characterization of Tpy-NH_2_ (ESI, Fig. S1†). In the range of 7.40–8.90 ppm, two doublets, one singlet and two triplets of the aromatic protons of the terpyridine (Tpy) moieties have appeared. In addition, the peaks in the 4.2–1.5 ppm range are attributed to aliphatic protons at the hexyl amine chain.

DDSQ functionalized with four epoxy end-groups (4EPX-DDSQ)^25^ is preferred to use as an initial compound due to the high reactivity of the oxirane rings. Telechelic DDSQ containing Tpy monomers (2Tpy-DDSQ) has been obtained after an epoxy-amine curing reaction between 4EPX-DDSQ and Tpy-NH_2_. In here, epoxy-amine curing reaction occurs *via* nucleophilic attach of the amine nitrogen on the terminal carbon of the epoxy function. According to Narracott, Chapman, Isaacs, and Parker, who are specialists on curing reaction, there are many possible reaction pathways occurring during the curing process.^27^ This indicates that generally a curing reaction is not selective. In the present study, curing reaction is just catalyzed by primer amine of NH_2_-Tpy (Scheme 1). No any additional catalyst has been used. Therefore it is believe that oxirane ring opening reaction occurs in moderate conditions, in here.

**Fig. 1:**
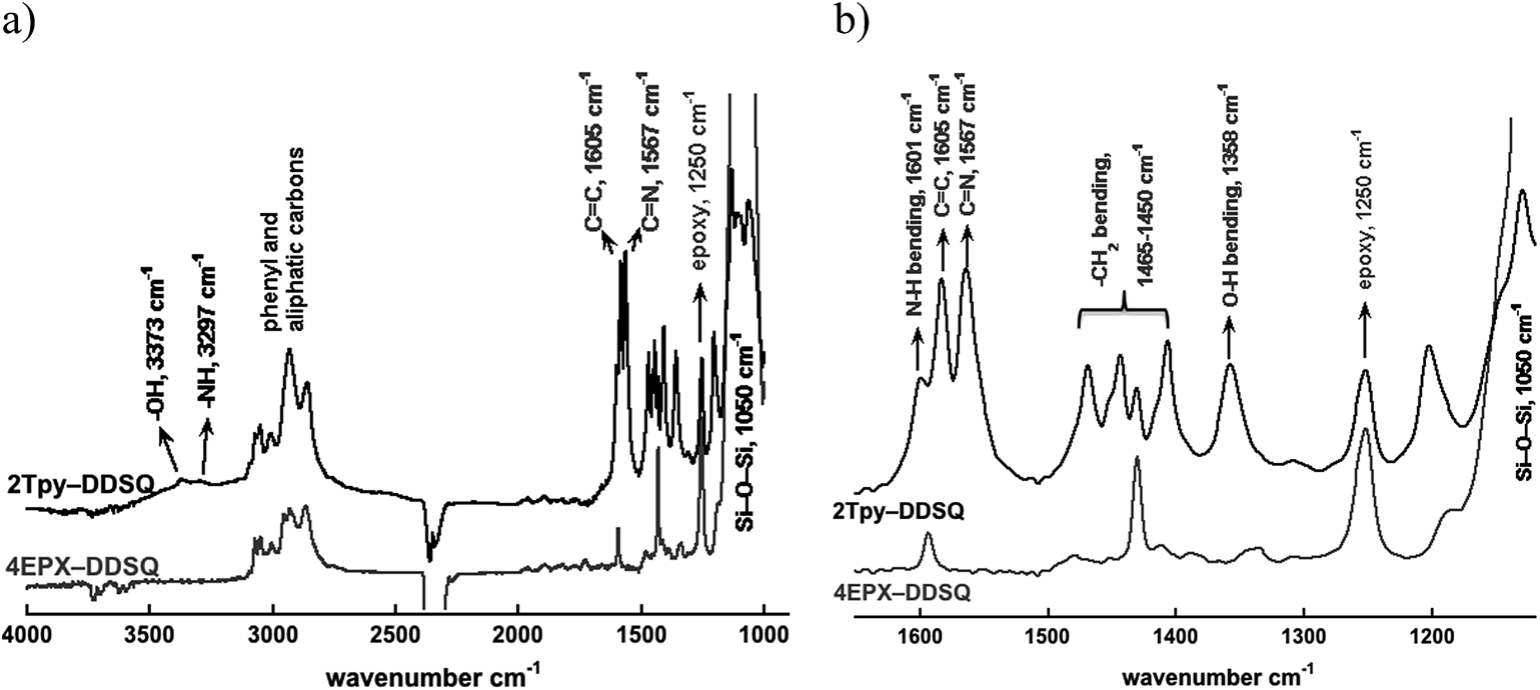
FTIR spectra of 4EPX-DDSQ (bottom) and 2Tpy-DDSQ (top), (a) in the 900–4000 cm^−1^ region, (b) in the 1100–1650 cm^−1^ region.

The structure of 2Tpy-DDSQ has been characterized by using FT-IR, UV-visible, NMR and MALDI-TOF/MS. Fig. 1 portrays the comparison between FT-IR spectra of 4EPX-DDSQ and 2Tpy-DDSQ. In the FT-IR spectrum of 2Tpy-DDSQ, the appearance of Si–O–Si absorbance at 1050 cm^−1^ indicates DDSQ core is preserved in the structure. Appearance of C=C absorbance at 1605 and C=N absorbance at 1567 cm^−1^ indicate that Tpy aromatic rings have been participated to the DDSQ structure. Observing of all these peaks in the FT-IR spectrum indicates successful connection of Tpy ligands to the DDSQ core. On the other hand, a strong vibration related to the ethylene oxide ring stretching is still appeared at 1250 cm^−1^ in the spectrum of 2Tpy-DDSQ, indicating some of ethylene oxide rings do not enter the reaction. NH and OH stretching frequencies are slightly observed respectively at 3297 and 3373 cm^−1^ (Fig. 1b), indicating secondary amine and hydroxyl groups have been generated after curing reaction. Moreover, N–H and O–H bending vibrations at 1601 cm^−1^ and at 1358 cm^−1^, respectively support the presence of secondary amine and hydroxyl groups. Consequently, according to the FT-IR results, the presence of stretching and bending vibrations related to secondary amine (N–H) have been confirmed. This result suggests that probably most of primary amines enter the reaction only once (Fig. 1a). According to the results taken from FT-IR, no further reaction could occur due to the moderate reaction condition.

**Fig. 2:**
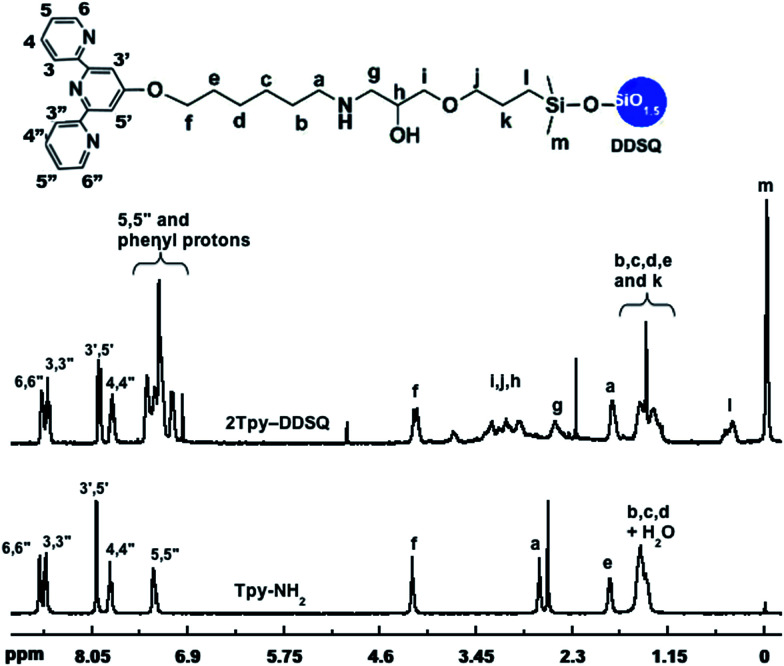
^1^H NMR of 2Tpy-DDSQ in CDCl_3_ (comparing with Tpy-NH_2_).

Upon comparison of the ^1^H NMR spectra of 2Tpy-DDSQ and Tpy-NH_2_, it is clearly seen that the resonances of the aromatic and methyl protons of Tpy-NH_2_ have appeared in the spectrum of 2Tpy-DDSQ even in the same ranges without any chemical shifting (Fig. 2). The integrations of the peak areas related to 2Tpy-DDSQ and Tpy-NH_2_ on the NMR spectra are given in ESI, Table S1.† Regarding the integration ratio of the peaks, it has been confirmed that two molecules of Tpy ligands are attached to one molecule of the DDSQ core by amine-epoxy curing reaction. On the other hand, the protons of unreacted ethylene oxide ring are overlapped with the protons of di(ethylene glycol) units attached to the DDSQ core. Therefore, the protons of unreacted ethylene oxide ring could not be observed separately in the NMR spectrum. As seen in the ESI, Table S1,† in case of the DDSQ arms (the one connected to Tpy ligand), the theoretical total numbers of protons are in very good agreement with the true integral values of these protons appeared in ^1^H NMR. This result is the main clue that clearly reveals the proposed structure, 2Tpy-DDSQ.

In order to provide more precise information about the molar mass and structure of the synthesized 2Tpy-DDSQ, MALDI-TOF/MS is presented in ESI Fig. S3.† Theoretical molecular weight of 2Tpy-DDSQ in which two Tpy ligands connect to one component of DDSQ core, is 2452. Therefore, the base peak at *m*/*z* = 2450.59 [M − 2] can be associated with the molecular ion peak of 2Tpy-DDSQ. Predict molecular weight of Tpy-DDSQ is approximately equal to results from mass spectrometry. The difference between theoretical molecular weight and base peak is probably attributed to loss of two hydrogen atoms shared by two nitrogen atoms maybe after ionization process of MALDI-TOF/MS.^26^

The aforementioned integration ratios obtained from ^1^H NMR and MALDI-TOF/MS are consistent and clearly verify the same structure, 2Tpy-DDSQ. In this structure, two Tpy ligands have attached to one DDSQ core. We believed that Tpy ligands have been attached to ethylene oxide rings that have diagonal position due to the steric hindrance effect of Tpy ligands. As mentioned above, the curing reaction composes of many reaction pathways. In this study, 2Tpy-DDSQ has been obtained in 20% yield after column chromatography. The structural characterizations of 2Tpy-DDSQ, especially ^1^H NMR, have been found satisfactory enough after being studied in detail as shown above. This achievement has remarkable importance before holding the complexation step.

### 3.2 Synthesis and characterization of the DDSQ based Ru(ii)-bis(terpyridine) complex, 2Tpy/Ru-DDSQ

In the first stage, according to the analysis results of ^1^H NMR and MALDI-TOF, the mole ratio of Tpy ligand to the DDSQ core is clearly confirmed to be a 2 : 1. In the second stage, 2Tpy-DDSQ has been coordinated a twofold excess amount of transition metal ions RuCl_3_·3H_2_O. This process yields a DDSQ based Ru(ii)-bis(terpyridine) complex, 2Tpy/Ru-DDSQ (Scheme 1). Coordination complex is soluble in various solvents and sufficiently stable to be processed and characterized. 2Tpy/Ru-DDSQ has been characterized by various methods, including FT-IR, NMR, XRD, electrochemical and photophysical analyses.

In the FT-IR spectrum of 2Tpy/Ru-DDSQ (ESI, Fig. S4†), some changes have been observed when comparing with the corresponding spectrum obtained from the starting reagent of 2Tpy-DDSQ. The main differences are based on the shifting of stretching vibrations of C=N (imino) and C=C bonds related to Tpy ring and NH bond attached to the DDSQ arms. The former one is associated to metal coordination. On the other hand in the latter case, the shifting of NH stretching to lower frequencies (3211 cm^−1^) is attributed to the increasing of the hydrogen bond ability of NH bonds.

**Fig. 3:**
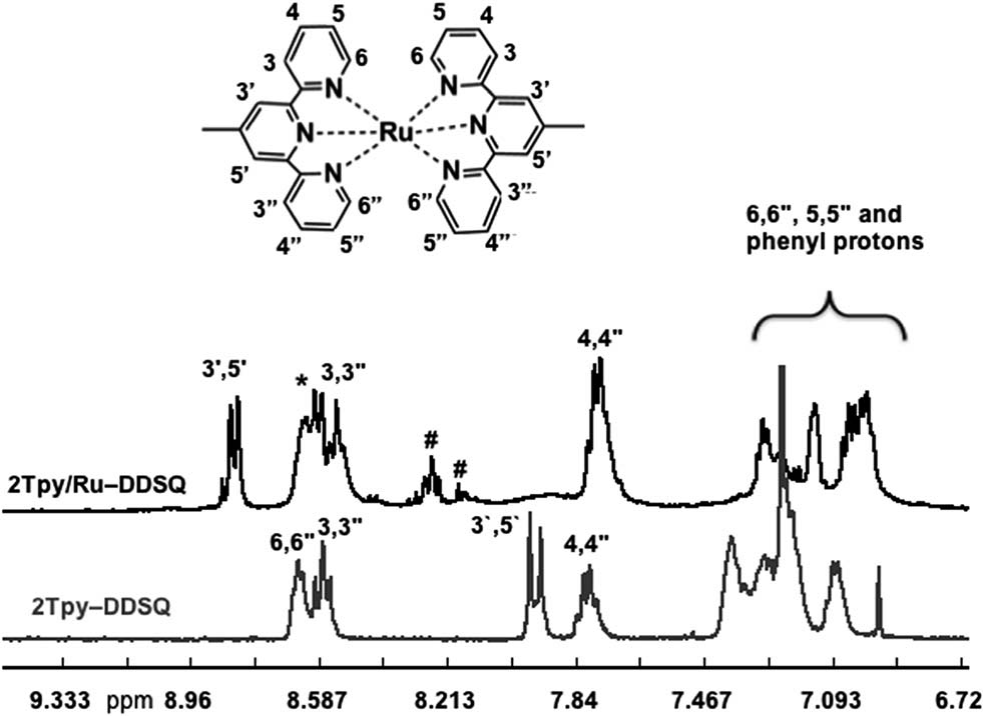
^1^H NMR spectra of 2Tpy-DDSQ (in CDCl_3_) and 2Tpy/Ru-DDSQ (in DMSO-*d*_6_) in the range of 6–9 ppm.

The complete ^1^H NMR spectrum of 2Tpy/Ru-DDSQ has been displayed in ESI, Fig. S5† and the range of aromatic protons related to [Ru(Tpy)_2_]^2+^ complex has been given in Fig. 3. As in the previous reports, after complexation reaction, the protons in 3′,5′ and 6,6′′ positions were shifted respectively to lower and higher fields due to the conformational change from the antiperiplanar to the synperiplanar.^28^ On the other hand, the aromatic protons of Tpy in 5,5′′ and 6,6′′ positions are overlapped with those of phenyl groups attached to the DDSQ core. The existence of aromatic proton nucleus and their chemical shifts are in agreement with the literature^29^ and demonstrate full complexation of the terpyridine ligands.

**Fig. 4:**
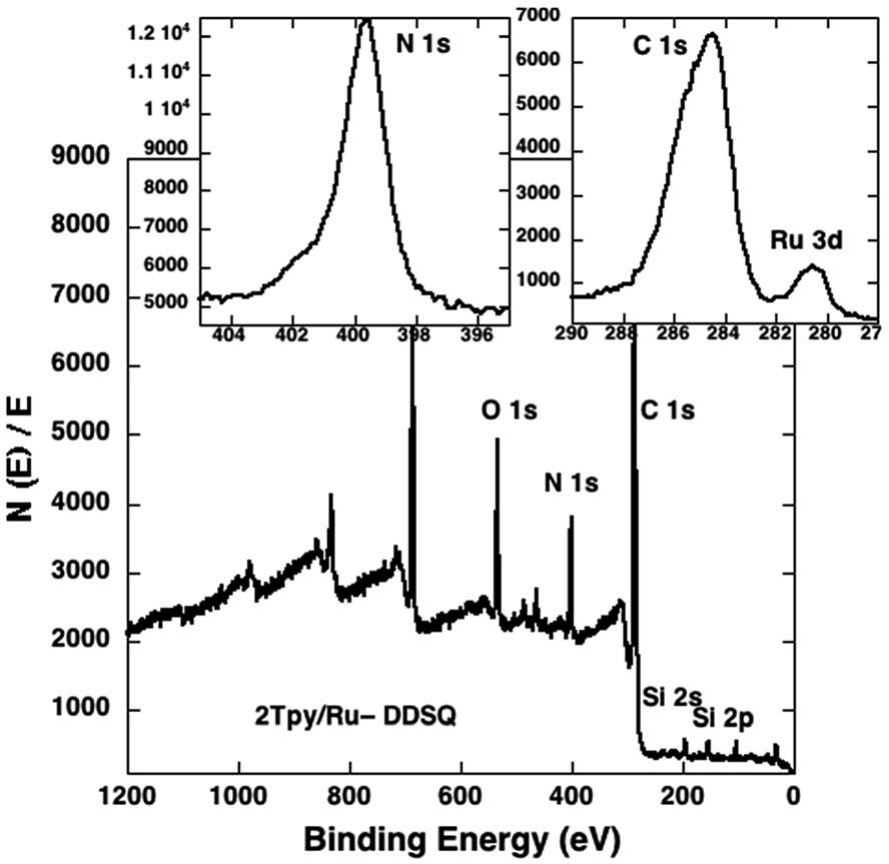
XPS spectrum of 2Tpy/Ru-DDSQ.

The composition of 2Tpy/Ru-DDSQ has been analysed using X-ray photoelectron spectroscopy (XPS). The XPS spectrum shows peaks at 534, 400, 288, 155, 102, and 281 eV which respectively indicate the presence of oxygen (1s), nitrogen (1s), carbon (1s), silicon (2s), silicon (1s) and ruthenium (3d) (Fig. 4). The presence of Ru signal on the XPS spectrum confirms expected complexation reaction and yields 2Tpy/Ru-DDSQ. Thermal properties of 2Tpy/Ru-DDSQ have been evaluated using TGA (ESI Fig. S7†). The initial weight loss for 4EPX-DDSQ occurs at 350 °C. On the other hand, 2Tpy/Ru-DDSQ shows reasonable stability, as the compound decomposes in air at 227 °C. The decreases for 2Tpy/Ru-DDSQ around 230 °C, 300 °C and 400 °C have been attributed to respectively decomposition of Tpy, organic segments around DDSQ core and DDSQ core itself.

### 3.3 Spectroscopy and photocurrent properties of 2Tpy/Ru-DDSQ

#### 3.3.1 UV-visible spectroscopy

**Fig. 5:**
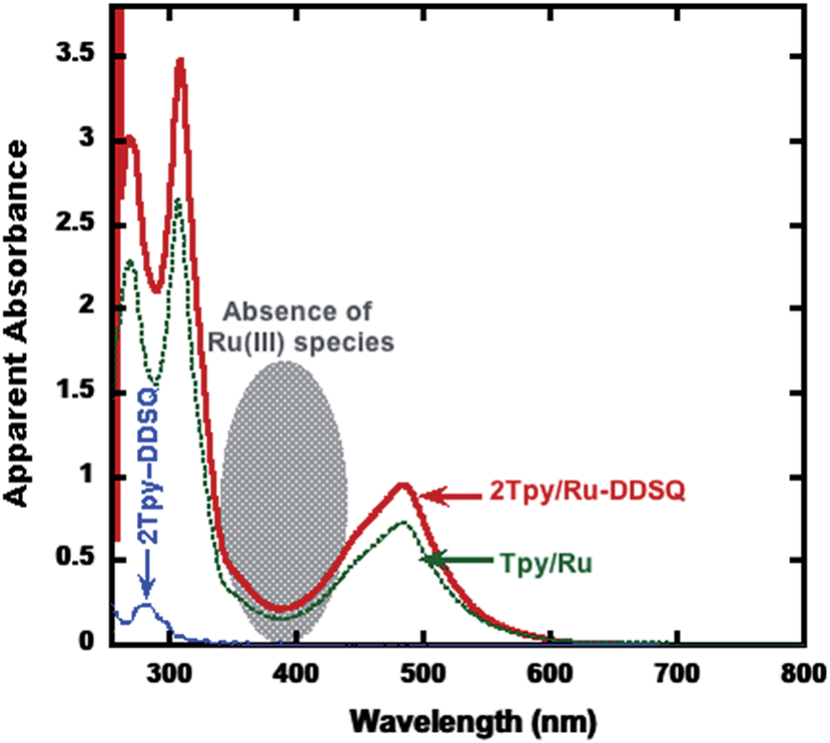
UV-vis absorption spectra of 2Tpy-DDSQ (dotted and blue line), 2Tpy/Ru-DDSQ (solid and red line), and the model compound Tpy/Ru (solid and dotted and green line) in acetone.

The optical properties of 2Tpy/Ru-DDSQ have been examined by UV-visible spectroscopy. Reddish solution of 2Tpy/Ru-DDSQ in acetone shows high- and low-energy absorption bands as seen in the Fig. 5. The high-energy absorption band at 280–300 nm is assigned to the intraligand (IL) [π → π*] transition of alkynyl (phenyl groups attached to the DDSQ core) and Tpy ligand, while the low energy absorption band at 400–480 nm is assigned to the metal-to-ligand charge-transfer (MLCT) for [Ru(Tpy)_2_]^2+^. On the other hand, the low-energy absorption bands at 481 nm with the increasing concentration range are found to obey Beer's law (ESI, Fig. S8†), indicating that there is no significant aggregation in acetone. To verify the assembly process of 2Tpy/Ru-DDSQ, polarized optical microscopy (POM) has been employed (ESI Fig. S9†). POM images of 0.01% and also 0.1% 2Tpy/Ru-DDSQ cast films display the homogenous dispersion without aggregation.

Normally, Ru(iii) species have absorption band around 400 nm. However, as seen in the Fig. 5 in shaded region, no any absorption band is available. Regarding this result together with complexation process (since a reducing agent, *N*-ethylmorpholine, has been utilized) all Ru(iii) are converted to Ru(ii).

As a further support for the structural confirmation of 2Tpy/Ru-DDSQ, a model compound (Tpy/Ru) based on [Ru(Tpy)_2_]^2+^ moiety is formed.^8^ The model compound Tpy/Ru is obtained through complexation of Tpy-NH_2_ with transition metal ions of RuCl_3_·3H_2_O in the presence of excess 2,2′:6′,2′′-terpyridine (Scheme S1, b†). A proportional increase in the light absorption ability of Tpy/Ru complex with increasing concentration (ESI, Fig. S2†) has confirmed the purity of Tpy/Ru. Since Tpy/Ru and 2Tpy/Ru-DDSQ complexes have the same optical species ([Ru(Tpy)_2_]^2+^), their light absorption characteristic and extinction coefficients should be the same^30^ (ESI Fig. S10†). The repeating unit of the coordination polymer contains a DDSQ core and a Ru(ii)-Tpy moiety. Therefore at the same intensity of absorbance (absorbance = *ε* × *c* × l), the concentration of Ru(ii)-Tpy moiety in coordination polymer should be the same with the concentration of Tpy/Ru. According to this assumption, the mole fraction of Ru(tpy)_2_^2+^ was determined to be 0.22 from the UV-vis absorption spectrum (see ESI Fig. S10, Appendix part†) indicating 22% Ru(ii)-Tpy moiety participate in the DDSQ nano building blocks.

**Chart 1:**
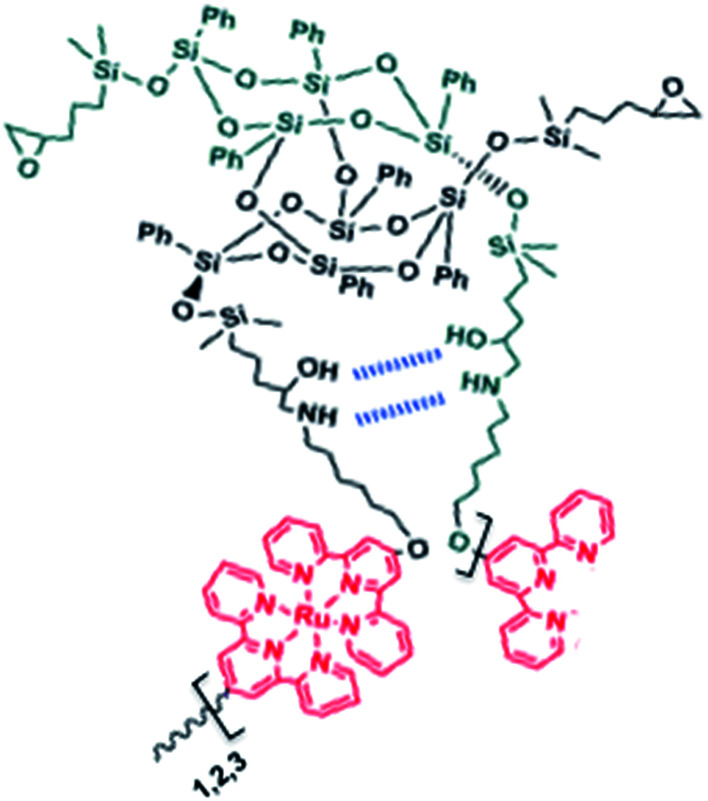
Proposed structure for 2Tpy/Ru-DDSQ.

As mentioned above, UV-visible spectroscopy of 2Tpy/Ru-DDSQ presents a characteristic MLCT band at 480 nm related to Ru(ii) bis(terpyridine) complexes, indicating full complexation. On the other hand, absence of an absorption band around 400 nm indicates that half complex of Ru(iii) has not been formed. In this condition, it can be assumed that full complexation with Ru(ii) occurs predominantly (Chart 1).

#### 3.3.2 Luminescence properties

**Fig. 6:**
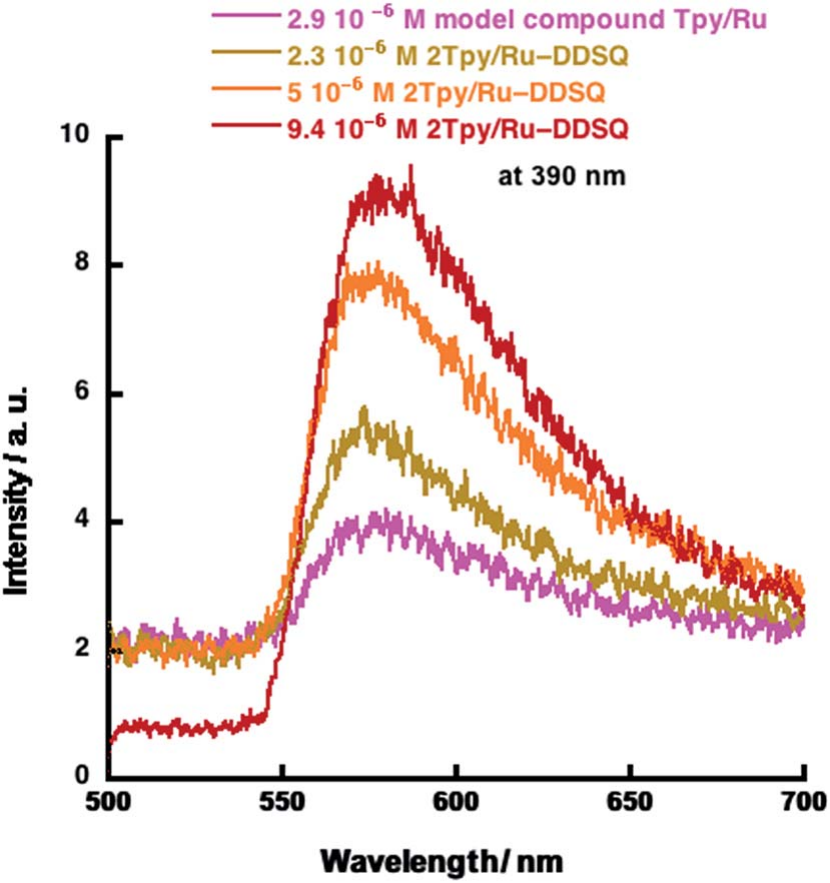
Emission spectra of the model compound Tpy/Ru and 2Tpy/Ru-DDSQ with different concentrations. Three different wavelengths of 390 nm, 400 nm and 410 nm are used as the excitation wavelengths for 2Tpy/Ru-DDSQ.

The emission spectra of 2Tpy/Ru-DDSQ and Tpy/Ru as measured in acetone, are depicted in Fig. 6. The emission spectrum is independent of the excitation wavelength since the same emission band (550–700 nm, a strong band at 610 nm) has been obtained using three different 390, 400, and 410 nm excitations. This band can be assigned to the Tpy-Ru complex, in agreement with the literature.^13^ On the other hand, as seen in the Fig. 6, the model compound Tpy/Ru and 2Tpy/Ru-DDSQ show very similar luminescence feature that is apparently attributed to absence of any negative effect of the DDSQ structure.

In the structure of 2Tpy/Ru-DDSQ, since the ratio of DDSQ core and [Ru(Tpy)_2_]^2+^ moiety is 1 : 1, the concentration of [Ru(Tpy)_2_]^2+^ moiety is about 50 mol%. In the literature, it is stated that concentration-based quenching occurs when the concentration of a metal ion complex is over 5 mol%.^31^ Although the fraction of [Ru(Tpy)_2_]^2+^ moiety in 2Tpy/Ru-DDSQ is about 22%, the luminescence intensity increases with increasing concentration of 2Tpy/Ru-DDSQ, indicating no emission quenching *via* electron transfer (Fig. 6). Despite the higher concentration of [Ru(Tpy)_2_]^2+^ moiety, it is believed that good assembling of [Ru(Tpy)_2_]^2+^ moiety in the coordination polymer possibly prevents the concentration-based quenching.

**Fig. 7:**
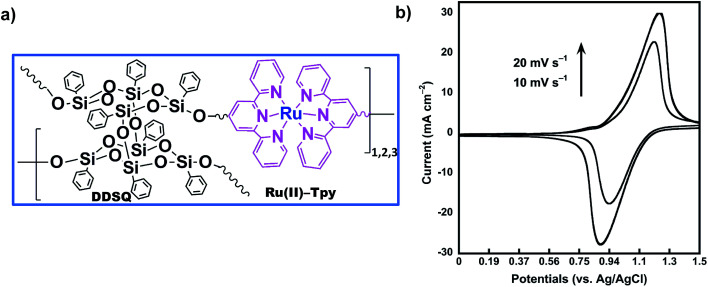
(a) Proposed structure of the coordination polymer 2Tpy/Ru-DDSQ. (b) Cyclic voltammograms for 2Tpy/Ru-DDSQ coated onto the ITO electrode at various scan rates.

Redox properties of the coordination polymer 2Tpy/Ru-DDSQ have been investigated using home-made cell containing 0.1 M KClO_4_ aqueous electrolyte. Fig. 7 portrays CV curves for ITO electrode modified by the 2Tpy/Ru-DDSQ cast film in the 0.1 M KClO_4_ electrolyte solution at a scan rate of 10 mV s^−1^ (Fig. 7a). The voltammogram respectively depicts well-defined surface waves consisting of almost symmetric oxidation at 1.21 V and reduction peaks at 0.9 V related to [Ru(Tpy)_2_]^2+^ moieties (Fig. 7b). Moreover, the height of current peaks increases linearly with the scan rates, showing immobilization of the redox species on the electrode.^32–34^ The electrochemical measurement indicates that electrochemical properties of [Ru(Tpy)_2_]^2+^ moieties are sustained in the coordination complex of the DDSQ structure.^35^

**Fig. 8:**
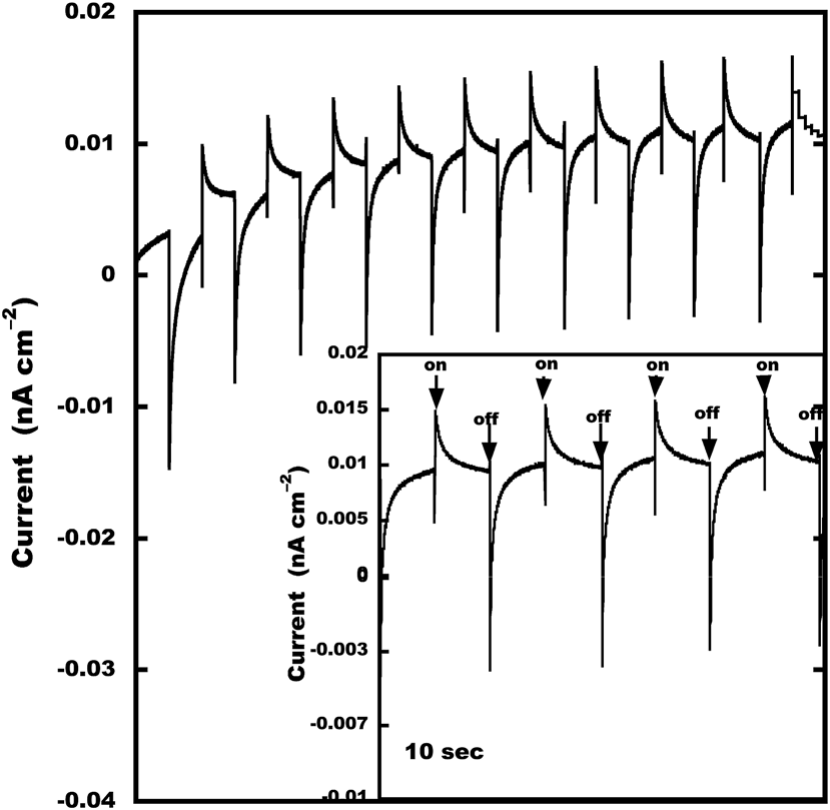
Photocurrent response of 2Tpy/Ru-DDSQ. Photocurrent was measured in a conventional three-electrode electrochemical cell with potential of 0 V *vs.* Ag/AgCl.

To understand the structure and optoelectronic property relationship of 2Tpy/Ru-DDSQ, an “on” and “off” photoswitchable photocurrent experiment has been conducted. A three-electrode method was used to study the photoelectric conversion property of 2Tpy/Ru-DDSQ coated on ITO under the illumination with a xenon lamp (*λ* = 450 nm, ESI Fig. S11†). The relationship between the photocurrent value and the irradiated time for 2Tpy/Ru-DDSQ is shown in Fig. 8. After the first few cyclings, photocurrent generation becomes relatively stable. Even in the absence of any external electron donor molecule in the degassed electrolyte solution, satisfactory anodic photocurrent efficiency (15 pA cm^−2^) has been obtained with the 2Tpy/Ru-DDSQ complex.^37^ This can be considered to be one of the clearest effects of DDSQ structure.

Generally photoexcited ruthenium complex [Ru(Tpy)_2_]^2+^* occurs when [Ru(Tpy)_2_]^2+^ moiety is excited by light. It is well known that [Ru(Tpy)_2_]^2+^* can be reduced and/or oxidized in the photoexcited state. Furthermore, in an anodic photocurrent process (reductive quenching), [Ru(Tpy)_2_]^2+^* can be quenched by an electron donor which transfers an electron to the [Ru(Tpy)_2_]^2+^* moiety. On the other hand, in the cathodic photocurrent process (oxidation quenching), [Ru(Tpy)_2_]^2+^* can be quenched by electron acceptor molecule where the electron transfer takes place from the [Ru(Tpy)_2_]^2+^* to the electron acceptor molecule.^36^ Thiol derivatives are well known as electron donor molecules.^37^ Similarly aliphatic amine molecules can serve as electron donor molecules owing to strong electron donating ability of the amino group.^38^ It is believed that amine quenches the chromophore by an electron transfer process, which forms an amine radical cation in a similar manner to thiol (ESI Fig. S12†). Then, termination of the couple radicals takes place. In this study, the amine groups nearby the [Ru(Tpy)_2_]^2+^ moiety in the structure of the coordination polymer 2Tpy/Ru-DDSQ execute the function of electron donor molecules. Since the required electron donor molecule for anodic photocurrent is already available in the structure, no additional electron donor molecule is needed.

**Fig. 9:**
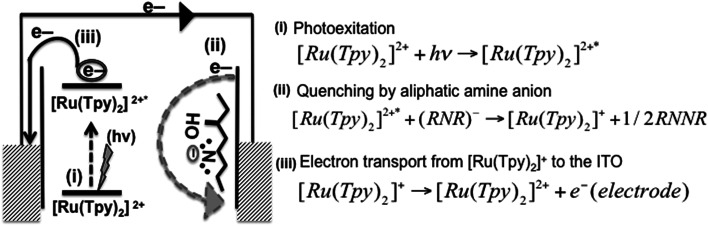
Proposed mechanism of the photoinduced electron-transfer reaction for 2Tpy/Ru-DDSQ.

As a mechanism for the photocurrent generation, first, the [Ru(Tpy)_2_]^2+^ chromophore is excited by light, and then the excited [Ru(Tpy)_2_]^2+^* chromophore is effectively quenched by the aliphatic amine anion in the same manner to thiols. The aliphatic amine anion turns into the [Ru(Tpy)_2_]^+^ species, which was confirmed in the previous studies.^39^ Then the anodic photocurrent is produced by the electron transport from the [Ru(Tpy)_2_]^+^ to the ITO electrode as seen in Fig. 9. The electron transport process is found to be acceptable due to the perfect assembling of [Ru(Tpy)_2_]^2+^ in the DDSQ nano building blocks.

## 4. Conclusions

To our knowledge, this is the first study in which a DDSQ based bis(terpyridine) Ru(ii) complex has been synthesized. Its structure has been clearly described by ^1^H NMR, XPS, UV-vis spectroscopy. It has been found that DDSQ based Ru(ii) complex offers many advantages including good thermal stability, and good solubility in common solvents, and high purity *via* column chromatography. On the other hand, CV measurement confirms the electrochemical stability of [Ru(Tpy)_2_]^2+^ moieties. Moreover, our results have been assisted by the investigation of the photocurrent response of 2Tpy/Ru-DDSQ. Efficient energy conversion obtained without the addition of a donor molecule is attributed to good intramolecular intercomponent interactions of [Ru(Tpy)_2_]^2+^ moieties in the DDSQ nano building blocks. Therefore, DDSQ based bis(terpyridine) Ru(ii) complex has a great potential to be used as a photosensitizer and deserves further studies.

## Conflicts of interest

There are no conflicts to declare.

## Supplementary Material

RA-008-C7RA12290J-s001
