# Supplementary material for: Synthesis and photochemical response of Ru(ii)-coordinated double-decker silsesquioxane
Source: RSC Adv. 2018 Jan 9;8(4):2148–56. doi: 10.1039/c7ra12290j (PMC9077204; doi:10.1039/c7ra12290j)
Supplement: RA-008-C7RA12290J-s001 [file RA-008-C7RA12290J-s001.pdf]

Electronic Supplementary Material (ESI) for RSC Advances.
This journal is © The Royal Society of Chemistry 2018

Synthesis and photochemical response of Ru(II)-coordinated double-decker silsesquioxane

Asuman Celik Kucuk[a*], Jun Matsui[b*], and Tokuji Miyashita[c]

[a]Metalurgical and Materials Engineering, Marmara University, Goztepe Campuse, 34722, Istanbul, Turkey

[b]Department of Material and Biological Chemistry, Yamagata University, 1-4-12, Kojirakawamachi, Yamagata 990-8560, Japan

[c]Institute for Multidisciplinary Research for Advanced Materials, Tohoku University, 2-1-1, Katahira, Aoba-ku, Sendai, 980-8577,

*Corresponding author: asuman.celik@marmara.edu.tr

*Co-Corresponding author: jun_m@sci.kj.yamagata-u.ac

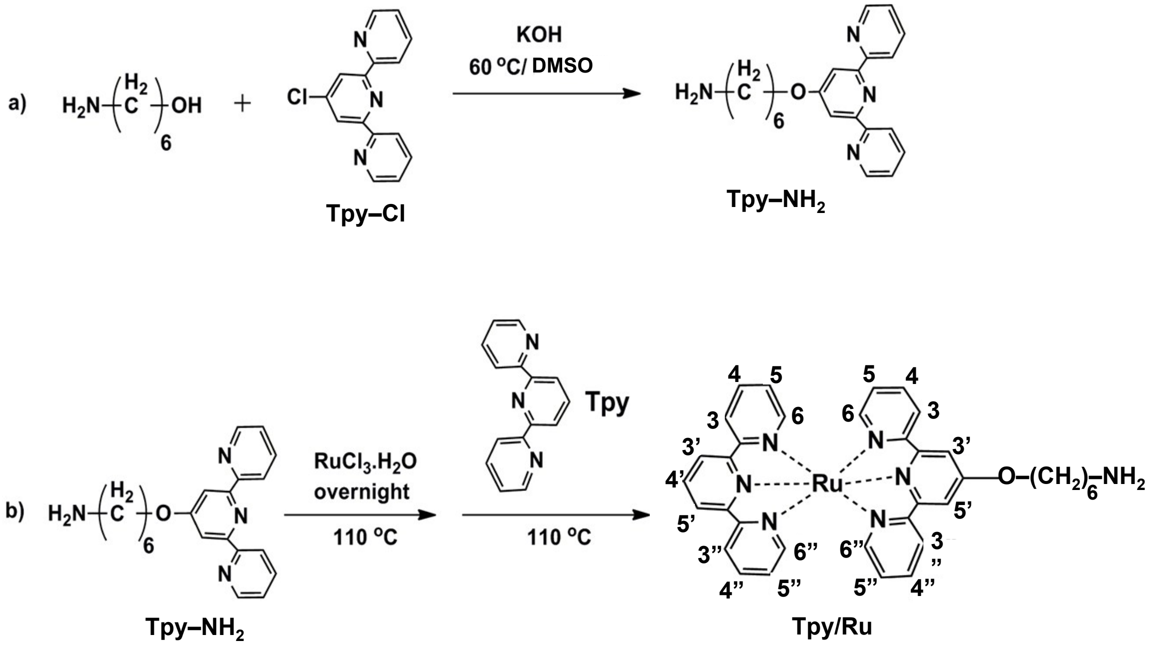

Scheme S1. Synthesis of amine functional terpyridine compound (Tpy-NH_2) (a) and model compound; Tpy/*Ru* (b).

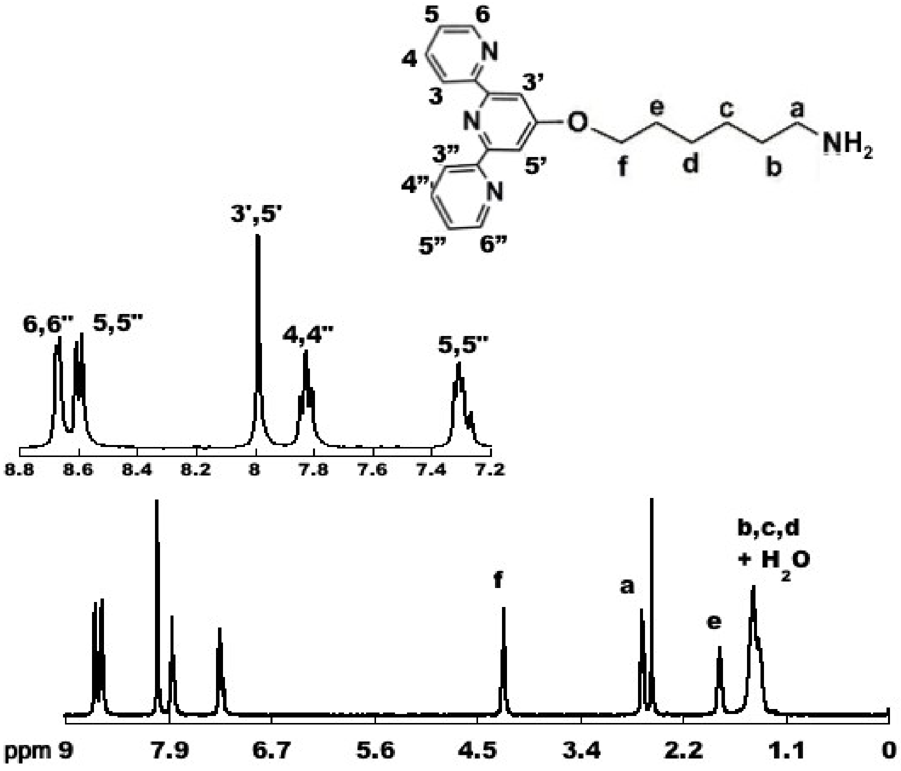

Figure S1. ^{1}H NMR spectrum of Tpy-NH_2.

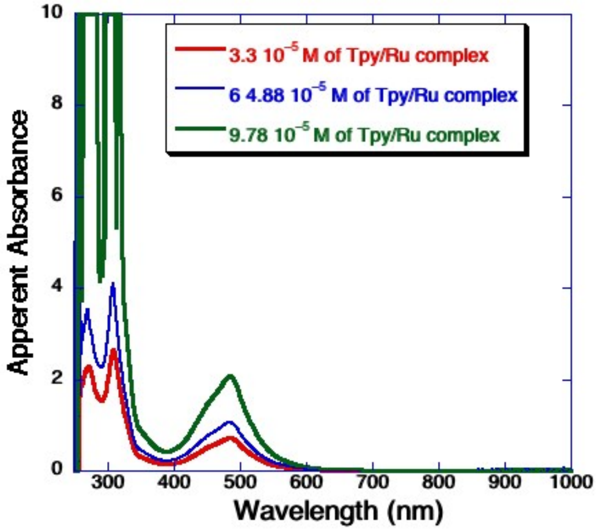

Figure S2. Concentration-dependent UV–vis absorption spectra of Tpy/Ru in acetone with increasing concentration at 298 K.

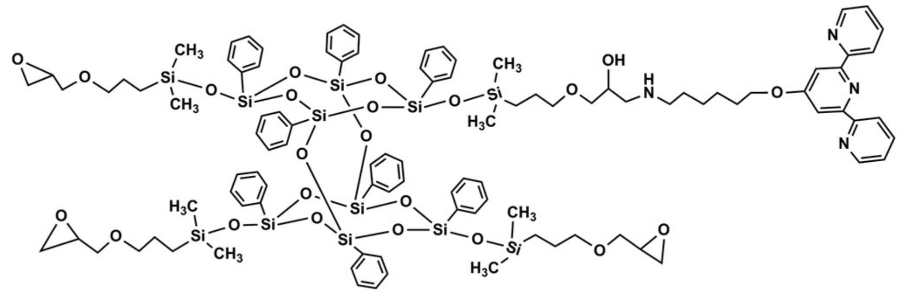

1Tpy-DDSQ
Chemical Formula: $C_{101}H_{132}N_4O_{23}Si_{12}$
Exact Mass: 2104,65

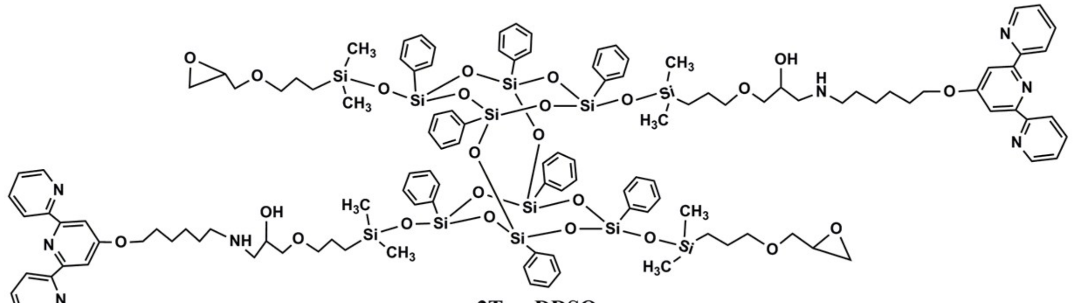

2Tpy-DDSQ
Chemical Formula: $C_{122}H_{156}N_8O_{24}Si_{12}$
Exact Mass: 2452,85

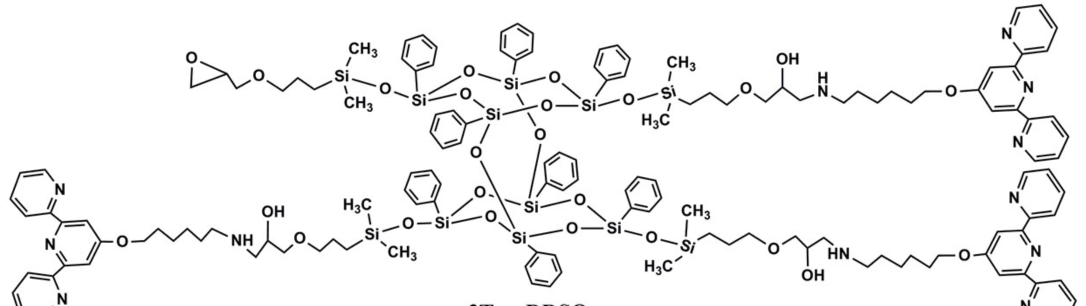

3Tpy-DDSQ
Chemical Formula: $C_{143}H_{180}N_{12}O_{25}Si_{12}$
Exact Mass: 2801,04

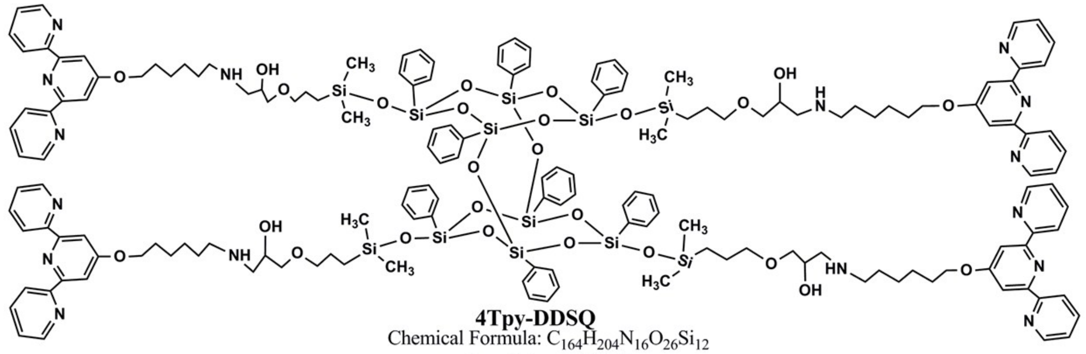

4Tpy-DDSQ
Chemical Formula: $C_{164}H_{204}N_{16}O_{26}Si_{12}$
Exact Mass: 3149,24

Chart S1: Possible products obtained from the epoxy-amine curing reaction.

Table S1. Comparing the theoretical number of protons at specific positions in the proposed molecule, 2Tpy–DDSQ, (for one arm of DDSQ) with the true value of the integral values of these protons appearing in ^{1}H NMR. Regarding the integration ratio of the peaks, it is found that two molecules of Tpy ligands are attached to one molecule of the DDSQ core.

**2Tpy–DDSQ**	m	l	k,b,c,d,e	j,i,h	g	a	f	Pyridine ring	Phenyl ring
Theoretic	6	2	10	5	2	2	2	4+2+2+2	20
Found	6	1.9	9.9	6.3	2.2	2.7	2	3.5+1.7+1.8+2.2	20.5

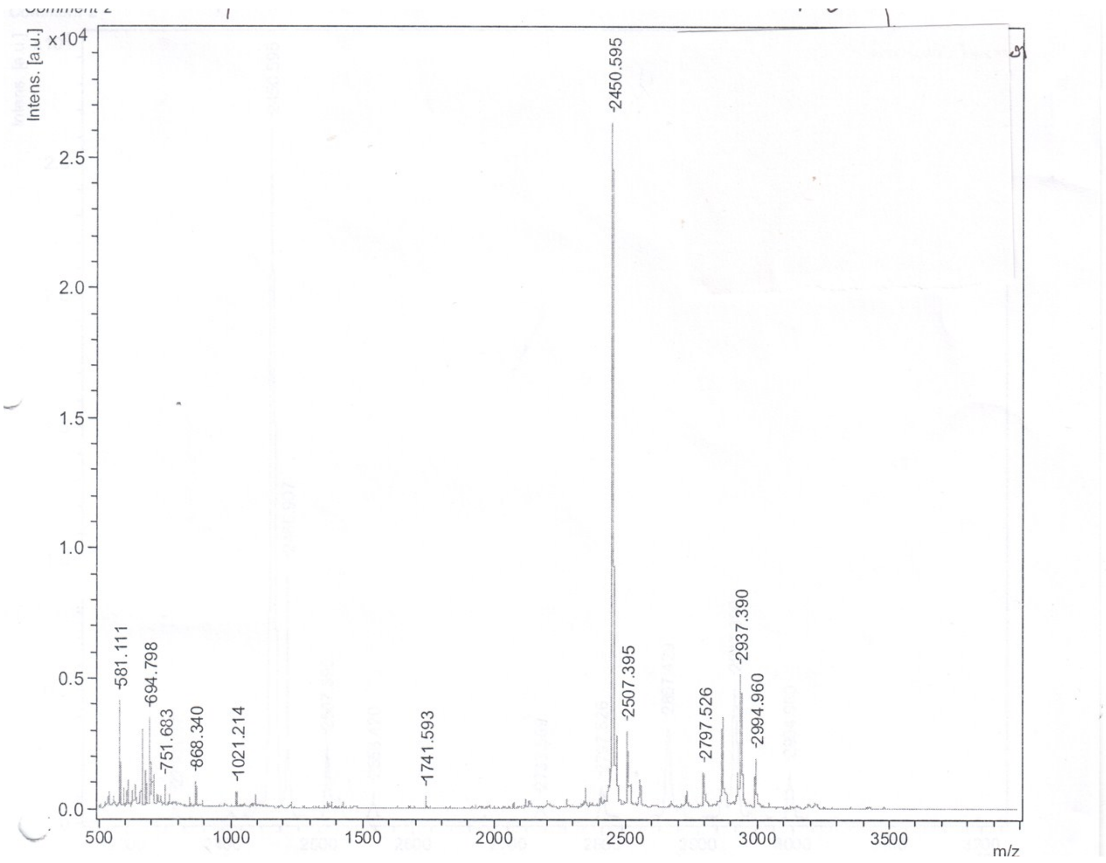

Figure S3. MALDI-TOF MS spectrum of 2Tpy–DDSQ.

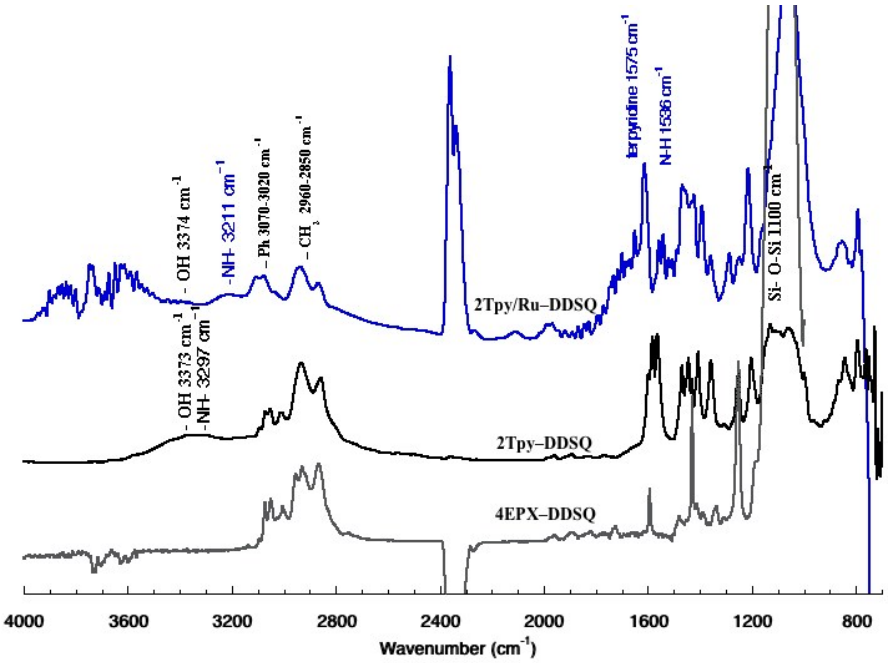

Figure S4. FTIR spectra of 4EPX–DDSQ (bottom), 2Tpy–DDSQ (middle) d 2Tpy/Ru–DDSQ (top) in the 750-4000 cm^{-1} ragion.

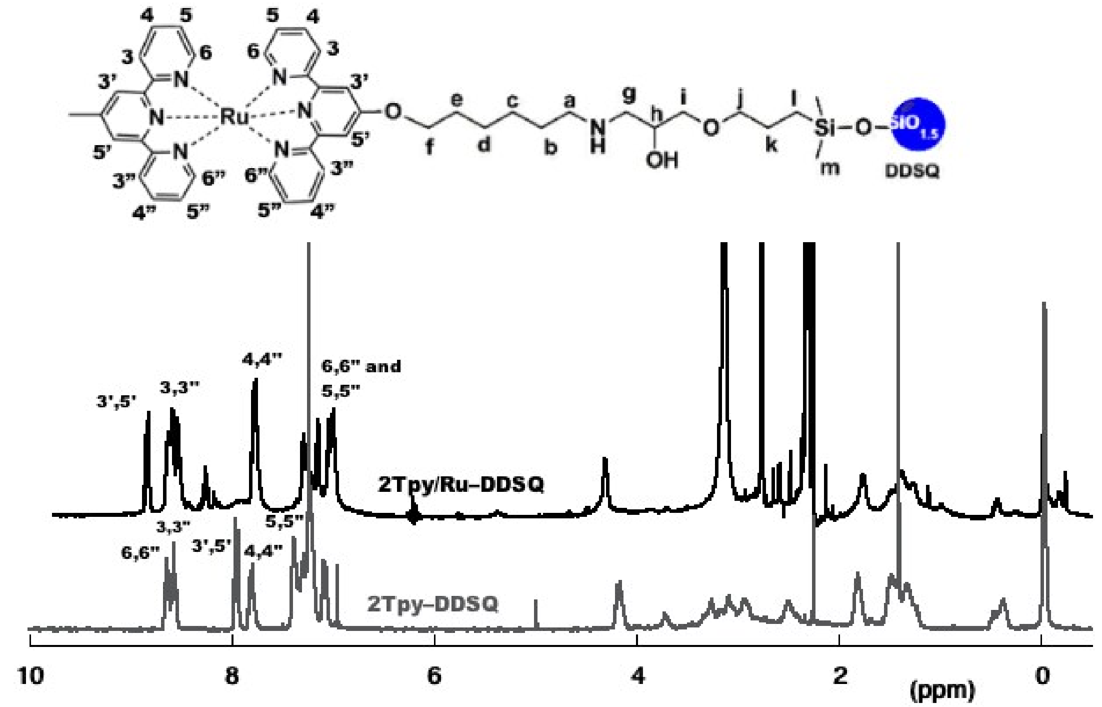

Figure S5. ^{1}H NMR spectra of 2Tpy–DDSQ (in $CDCl_3$) and 2Tpy/*Ru*–DDSQ (in DMSO-d_6).

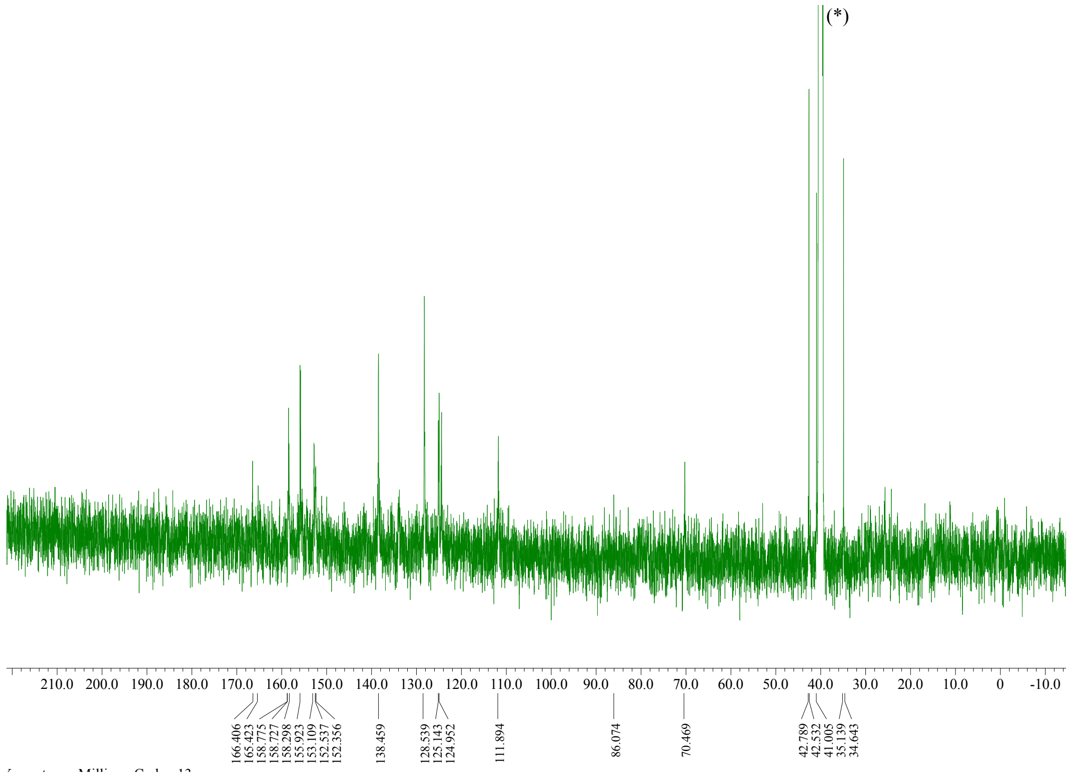

Figure S6. ^{13}C NMR spectrum of 2Tpy/*Ru*–DDSQ in DMSO-d_6. (* is related to DMSO)

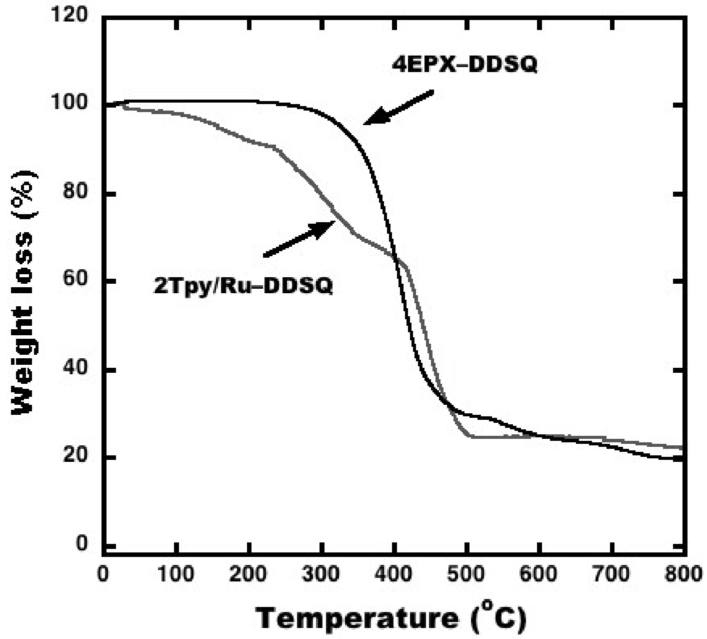

Figure S7. TGA thermograms of 4EPX–DDSQ and 2Tpy/Ru–DDSQ in air atmosphere.

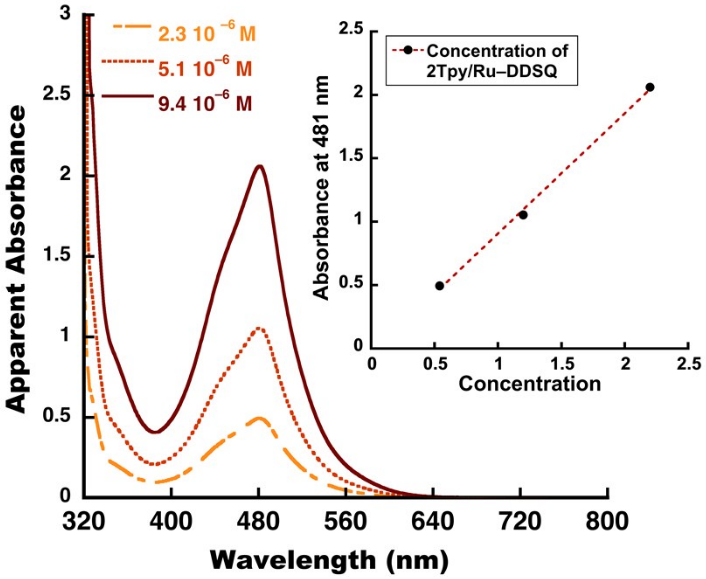

Figure S8. Concentration-dependent UV–vis absorption spectra of 2Tpy/Ru-DDSQ in acetone with increasing concentration at 298 K. The inset shows the apparent absorbance at 481 nm as a function of concentration of 2Tpy/Ru-DDSQ,

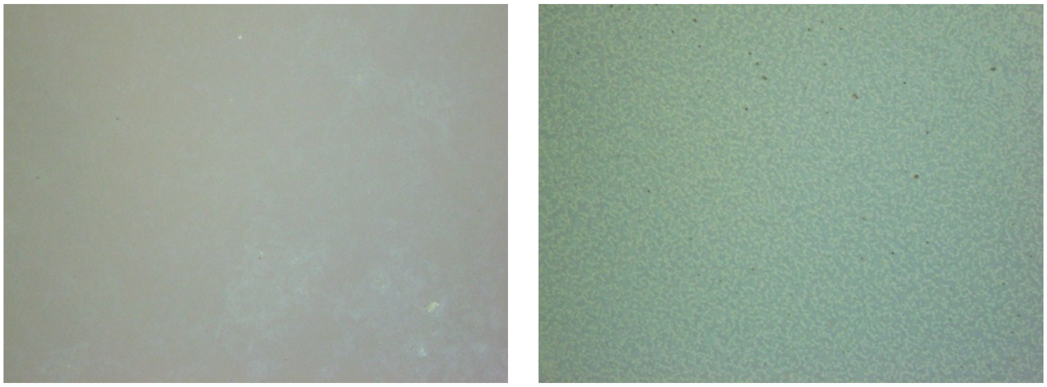

Figure S9. POM images of (a) 0.01% (b) 0.1% cast films of 2Tpy/Ru–DDSQ obtained from acetone solution.

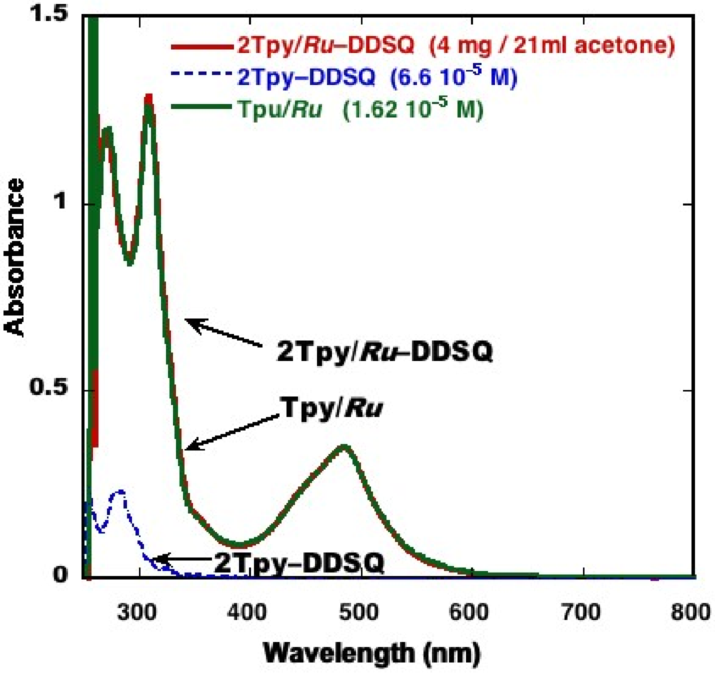

Figure S10. Absorption spectra of 2Tpy/Ru–DDSQ and Tpy/Ru show the dependency of light absorption characteristics on the concentration.

**Appendix:** Mole fraction of Ru(II)-Tpy moiety incorporated in **2"Tpy/Ru-DDSQ according to UV-vis spectroscopy (Figure S10).**

Solution preparation of 2Tpy/Ru-DDSQ for UV-vis spectroscopy:
0.004 g of 2Tpy/Ru-DDSQ was added in 21 ml acetone. The concentration of Ru(II)-Tpy moiety in coordination complex should be the same with that of model compound Tpy/*Ru*. *(M=1.62x10^{-5} mol/L).* So the mole of Ru(II)–Tpy moiety in the DDSQ nano building blocks is as following

$$M = mol / Volume$$

$$1.62 \times 10^{-5} = \aleph / 0.021$$

$\aleph = 3.4 \times 10^{-7} mol$ of Ru(II)–Tpy (A: mole of Ru(II)/Tpy moiety

The MW of telechelic DDSQ–Tpy monomer, 2Tpy–DDSQ, is obtained from Maldi-TOFF as 2450 g/mol. Moreover, the mole of Ru(II)–Tpy moiety in 2Tpy/Ru–DDSQ is found as 3.4×10^{-7}.

__**Lets think about the theoretical mole of Ru related to Ru(II)-Tpy.**__
If the repeating unit is n, 1 mole 2Tpy/Ru–DDSQ is n×2551 gram according to the following equation.

$$1mol(2Tpy / Ru - DDSQ) = nmol(Ru) + nmol(2Tpy - DDSQ)$$

$$1mol(2Tpy / Ru - DDSQ) = n \otimes 101g + n \otimes 2450g = n \otimes 2551g$$

In the case of full complex of 2Tpy-DDSQ, in (n)×(2551) g 2Tpy/Ru–DDSQ, there is (n)×(101) gram Ru. Since we used 0.004 g *2Tpy/Ru–DDSQ, the gram of Ru 2Tpy/Ru–DDSQ is as following;*

$$\frac{[0.004g2Tpy / Ru - DDSQ] \otimes [n \otimes 101gRu]}{n \otimes 2551gram2Tpy / Ru - DDSQ} = 1.583 \times 10^{-4} gramRu$$

If the total mass of Ru related to Ru(II)-Tpy is 1.583 x 10^{-4} gram, the total mole of Ru would be as following:

$$n = m / MW$$

$$n_{Ru} = \frac{1.583x10^{-4} g}{101g / mol} = 1.57x10^{-6} mol \Rightarrow B$$

If A is the exact mole of Ru(II)-Tpy moiety according to the UV measurement and B is the theoretical mole of Ru(II)-Tpy,

A/B indicates that

$$\frac{3.4x10^{-7} mol_{Ru} - in - coordination - polymer}{1.57x10^{-6} total - mol_{Ru}} = 0.22 \Rightarrow 22\%$$

22% Ru(II)–Tpy moiety participate in 2Tpy/Ru–DDSQ.

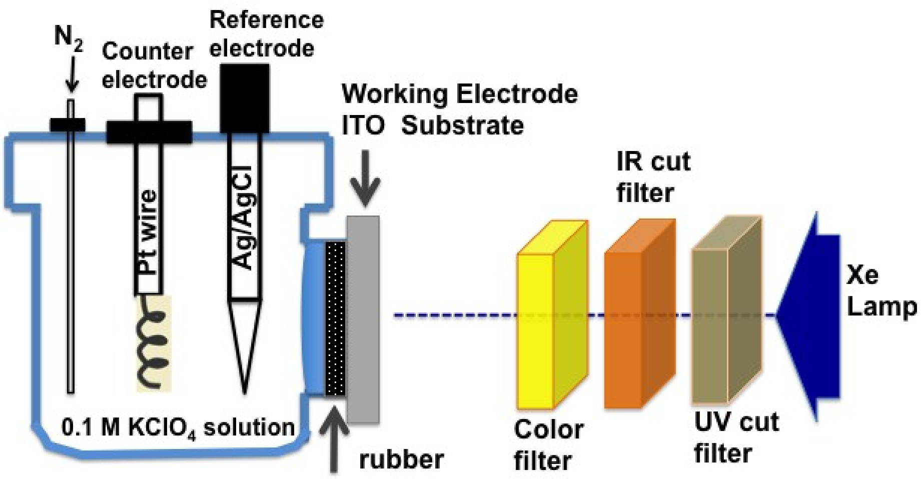

Figure S11. Schematic configuration of photocurrent measurement.

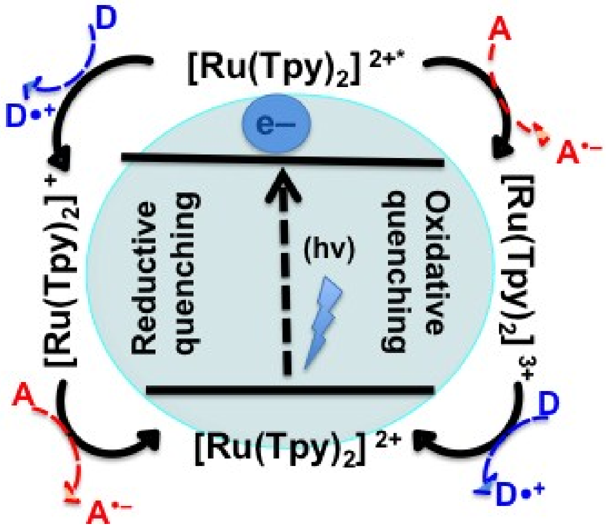

Figure S12. Proposed photoinduced electron transfer reactions.
